# Quantification of Hand Motor Symptoms in Parkinson’s Disease: A Proof-of-Principle Study Using Inertial and Force Sensors

**DOI:** 10.1007/s10439-017-1881-x

**Published:** 2017-07-19

**Authors:** Josien C. van den Noort, Rens Verhagen, Kees J. van Dijk, Peter H. Veltink, Michelle C. P. M. Vos, Rob M. A. de Bie, Lo J. Bour, Ciska T. Heida

**Affiliations:** 1 Biomedical Signals and Systems Group, MIRA Research Institute for Biomedical Technology and Technical Medicine, University of Twente, P.O. Box 217, 7500 AE Enschede, The Netherlands; 2 Department of Neurology and Clinical Neurophysiology, Academic Medical Center, Amsterdam, The Netherlands; 3 Department of Rehabilitation Medicine, VU University Medical Center, Amsterdam Movement Sciences, Amsterdam, The Netherlands; 4 Department of Radiology and Nuclear Medicine, Musculoskeletal Imaging Quantification Center, Academic Medical Center, Amsterdam Movement Sciences, Amsterdam, The Netherlands

**Keywords:** Parkinson’s disease, Hand, Fingers, Bradykinesia, Tremor, Rigidity, Inertial sensors, Movement analysis

## Abstract

This proof-of-principle study describes the methodology and explores and demonstrates the applicability of a system, existing of miniature inertial sensors on the hand and a separate force sensor, to objectively quantify hand motor symptoms in patients with Parkinson’s disease (PD) in a clinical setting (off- and on-medication condition). Four PD patients were measured in off- and on- dopaminergic medication condition. Finger tapping, rapid hand opening/closing, hand pro/supination, tremor during rest, mental task and kinetic task, and wrist rigidity movements were measured with the system (called the PowerGlove). To demonstrate applicability, various outcome parameters of measured hand motor symptoms of the patients in off- vs. on-medication condition are presented. The methodology described and results presented show applicability of the PowerGlove in a clinical research setting, to objectively quantify hand bradykinesia, tremor and rigidity in PD patients, using a single system. The PowerGlove measured a difference in off- vs. on-medication condition in all tasks in the presented patients with most of its outcome parameters. Further study into the validity and reliability of the outcome parameters is required in a larger cohort of patients, to arrive at an optimal set of parameters that can assist in clinical evaluation and decision-making.

## Introduction

Parkinson’s disease (PD) is an age-related neurodegenerative disorder, second in prevalence to Alzheimer’s disease.^4^ In current practice, the severity of the motor symptoms that partially defines the clinical condition of a PD patient is scored during a standardized neurological examination, using the motor examination part of the unified Parkinson’s disease rating scale (UPDRS-ME).^9^,^14^

Assessment of hand movements is an important part of the UPDRS-ME and includes items for bradykinesia, hand tremor and wrist rigidity.^8^^–^ 10,19,27 These are symptoms that strongly respond to dopaminergic medication and deep brain stimulation (DBS) and are therefore often used to judge the effects of these therapies. However, the assessment of the corresponding movements may often vary between clinicians and depends on the level of experience of the neurologist or movement disorder nurse. This subjective nature introduces extra variability to the UPDRS-ME.^27^,^28^,^30^,^31^,^34^ Furthermore, the current clinical exam may not be able to detect small changes as all items are scored on a five-point scale.^8^,^27^,^30^,^31^ A single scoring may be dependent on multiple measures such as speed, amplitude, decrease in amplitude over time, and occurrence of hesitations. To adequately study the PD motor symptoms including the effects of medication or DBS and the fluctuations over a period of time, it is worthwhile to find an objective and quantified measure of (the specific components of) these symptoms.

Objective quantification of PD hand motor symptoms has been the subject of several studies (examples are given in Table 1). However, previous studies had some limitations since their systems were either very complex (e.g. Ref. 26,29), unable to measure all hand motor symptoms in one assessment (e.g. Ref. 11,12,16,24,31), or not applied in patients (e.g. Ref. 3). Therefore, these systems are not easily available and/or often clinically not applicable.

**Table 1:** Examples of studies using objective quantification of PD motor symptoms.

Study	Measurement system	Placement on body	Parameters
Bradykinesia			
Dunnewold *et al.* 5	Accelerometer	Wrist	Mean acceleration Tap rate Time of movement interval
Tavares *et al.* 36	Quantitative digitography using a computerized-interfaced keyboard	Not applicable	Time of movement interval Velocity Frequency
Koop *et al.* 20	Gyroscope	Hand	Root mean square angular velocity
Keijsers *et al.* 19	Accelerometer	Several body parts	Mean velocity
Salarian *et al* 32	Gyroscope	Forearm	Root mean square angular velocity Amplitude Total range of motion
Tabbal *et al.* 35	Gyroscope	Forearm	Root mean square angular velocity
Jun *et al.* 18	Gyroscope	Hand	Root mean square angle and angular velocity Peak power and total power of angular velocity
Endo *et al.* 8	Magnetic sensor consisting of two coils	Thumb and tip of index finger	Maximum velocity Amplitude of position data Time of movement interval
Pulliam *et al.* 31	Accelerometer and gyroscope	Index finger	Amplitude Velocity Rhythm
Mentzel *et al.* 23	Accelerometer and gyroscope Magnetometers	Forearm Upper arm Waist	Time of movement interval Amplitude Velocity
Djuric-Jovicic *et al.* 3	Gyroscopes	Thumb and tip of index finger	Time of movement interval Angular velocity Total angular range of motion (aperture)
Tremor			
Keijsers *et al.* 19	Accelerometer	Several body parts	Percentage peak frequencies above 4 Hz
Salarian *et al.* 32	Gyroscope	Forearm	Root mean square angular velocity Amplitude Total range of motion
Giuffrida *et al.* 12	Accelerometer and gyroscope	Middle finger	Peak power acceleration and angular velocity Frequency of the peak power Root mean square of angular velocity and of angle
Gallego *et al.* 11	Gyroscope	Forearm Hand	Amplitude angular velocity Frequency
Mostile *et al.* 24	Accelerometer and gyroscope	Middle finger	Root mean square amplitude Peak power angular velocity and acceleration
Heldman *et al.* 16	Accelerometer and gyroscope	Index finger	Peak power of angular velocity
Scanlon *et al.* 33	Accelerometer	Upper and lower limbs	Root mean square of acceleration
Daneault *et al.* 1	Accelerometer (in smart phone)	Unknown	Maximum amplitude acceleration Peak power, median power and power distribution of acceleration
Pulliam *et al.* 31	Accelerometer and gyroscope	Index finger	Amplitude
Rigidity			
Prochazka *et al.* 30	Hand-held force sensing device (air-filled pads) combined with a compliant displacement gauge	Wrist	Impedance Viscous damping constant Elastic stiffness
Fung *et al.* 10	Torque motor Electromyography	Forearm Hand	Impulse Work
Patrick *et al.* 27	Pads connected to a force transducer Gyroscope	Elbow Wrist	Elastic stiffness Viscous damping constant Impedance
Sepheri *et al.* 34	Test rig with strain gage force transducer and potentiometer	Elbow	Range of motion Viscous damping constant Elastic stiffness
Tabbal *et al.* 35	Pads connected to a force transducer Gyroscope	Elbow Wrist	Impedance
Park *et al.* 26	Torque motor Potentiometer Load cell Accelerometer	Wrist	Torque Viscous damping constant Elastic stiffness Work Impulse
Endo *et al.* 7,8	Force sensor Gyroscope Electromyography	Wrist	Torque Elastic coefficient
Powell *et al.* 29	Torque motor Electromyography	Wrist	Work Impulse Reflex responses
Kwon *et al.* 22	Load cell Potentiometer Accelerometer	Wrist	Viscous damping constant Elastic stiffness Impulse Impedance

In this study, an alternative system to obtain accurate hand and finger kinematics in PD patients is proposed, called the PowerGlove system, which is a combination of miniature inertial and magnetic sensors on each finger segment and the back of the hand.^21^ Combination of these sensors enables a 3D reconstruction of the movements of all finger joints and the orientation of the hand. In combination with a force sensor, also joint rigidity could be quantified. Therefore, the PowerGlove might have potential to be used for clinical research into PD motor symptoms without extensive changes to the clinical setting. With this, the effect of dopaminergic medication or DBS could potentially be evaluated.

For application, the system should meet certain requirements, which firstly include clinical applicability measuring all hand motor symptoms in one clinical assessment, and a good validity on its outcome parameters to differentiate between certain conditions. Other requirements are further validation to discrimination between different clinical scores and a good intra- and inter-reliability. Measurement of hand and finger kinematics with the PowerGlove has already been evaluated with an optoelectronic motion capture system as a reference system.^37^ However, application of the PowerGlove for quantification of PD hand motor symptoms in different conditions has not been studied yet.

Therefore, in this proof-of-principle study we explore and demonstrate the applicability of quantifying hand motor symptoms in PD patients with the PowerGlove and an additional force sensor in a clinical setting (in which the patients are admitted to the hospital for a DBS surgery screening and assessed in off- and on- dopaminergic medication condition). This study is part of a larger ongoing project to test the validity and reliability of outcome parameters in a larger cohort of PD patients. In this paper, we first aim to describe the methodology of the application, and demonstrate the results of all hand motor symptoms in a few representative PD patients in off- vs. on- dopaminergic medication condition, by means of measured differences in multiple outcome parameters.

## Methods

### Subjects

Four PD patients were recruited from the Academic Medical Center (AMC) in Amsterdam, the Netherlands, where they were admitted for an extensive two-day screening in order to become candidates for DBS surgery. In this proof-of-principle study, data of these 4 PD patients (selected randomly from a larger cohort), with a difference of at least 1 point in at least one of the UPDRS-ME hand items in off- vs. on-condition, are presented to demonstrate multiple outcome parameters of the measurement system. Inclusion criteria were occurrence of PD symptoms for more than five years, which are interfering with daily life activities. Patient showed in general a good response to dopaminergic medication but suffered from dopamine-dependent motor response fluctuations with or without levodopa induced dyskinesia. Furthermore, patients had to be able to communicate adequately in Dutch or English, and be older than 18 years. Exclusion criteria were a medical history other than PD which restricted hand movements and an inability to correctly place the PowerGlove sensor units on the patient’s hand or to correctly perform anatomical calibration (described below). The study was approved by the local Medical Ethical Committee. Full written informed consent was obtained from all patients.

### PowerGlove System

**Figure 1:**
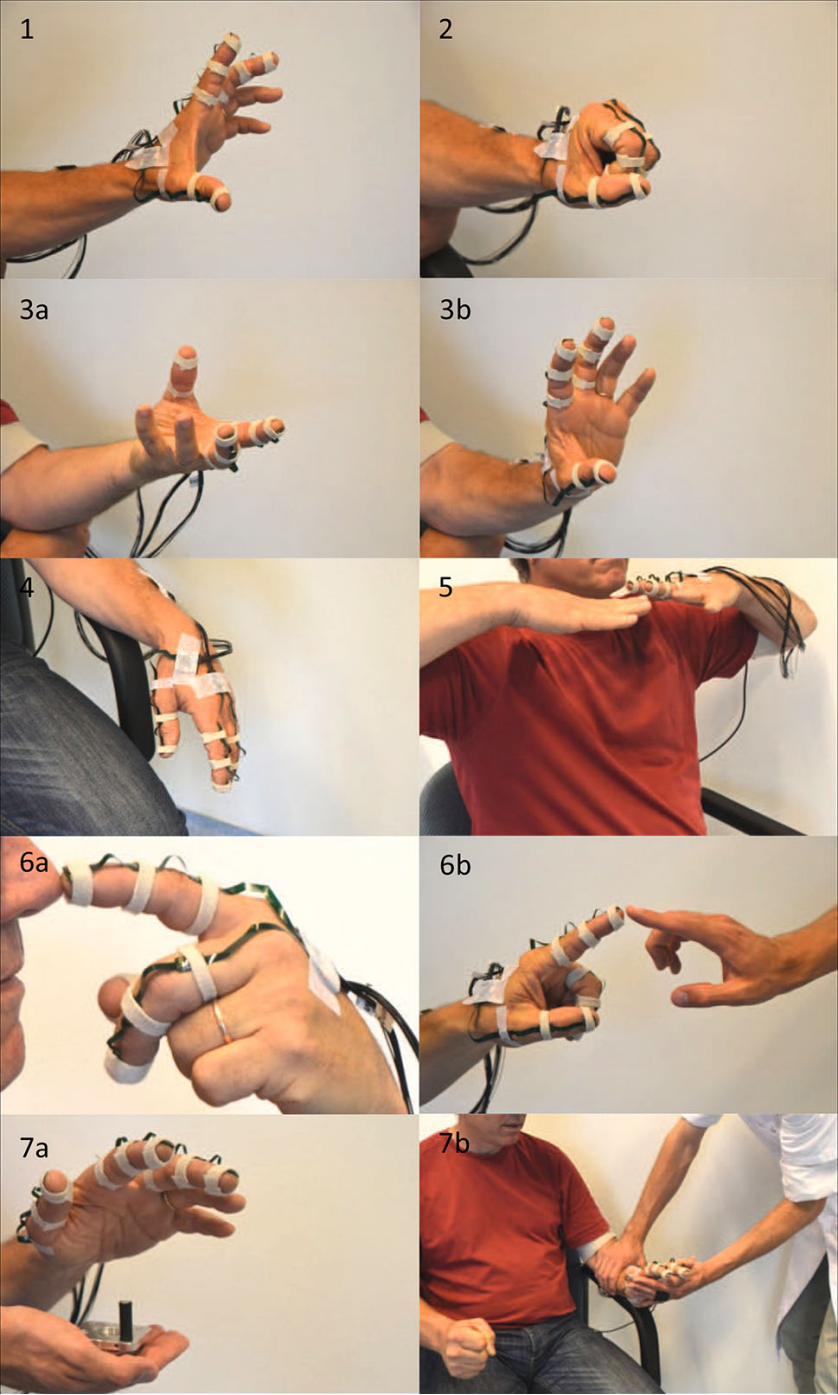
Assessment of hand motor symptoms in a patient with Parkinson’s disease, using the PowerGlove (miniature inertial sensors on the hand and fingers and, for rigidity assessment, an additional force sensor on the palmar side of the hand). From left to right and top to bottom: (1) rapid finger tapping (thumb/index), (2) rapid hand opening/closing movements and (3a, b) pro/supination of the hand for assessment of bradykinesia; tremor was assessed (4) during rest with and without a mental task, (5) during a posturing task (holding hands outstretched below chin) and (6a, b) during an active kinetic task (moving index finger between patient’s nose and the finger of the examiner); (7a, b) the wrist rigidity test consisted of passive wrist flexion/extension (performed by the examiner) with and without contralateral activation by making a fist.

Eleven sensor units^21^ were attached to the dorsal side of the hand and fingers; on the metacarpal, proximal and distal phalanges of the thumb and the proximal, middle and distal phalanges of the index and middle fingers using small Velcro straps (Fig. 1). Two sensor units were taped on the back of the hand, of which the one connected to the index and middle finger string was used for the hand orientation. An additional sensor was attached to the dorsal side of the lower arm to be able to measure wrist kinematics. All sensor units contained a 3D gyroscope and a 3D accelerometer (ST LSM330DLC). Additionally, the sensor units on the distal phalanges, metacarpal of the thumb, back of the hand and the lower arm also contained a 3D magnetometer (Honeywell HMC5983).

### Clinical PD Motor Symptom Assessment

For each patient, PD motor symptoms were measured in off-medication condition, in the morning after overnight withdrawal from dopaminergic medication; and afterwards in on-medication condition, at the time of optimal medication effect, typically 1 h after medication intake (120% of normal morning dose). At both times, the patient had a clinical evaluation by a nurse specialized in movement disorders guided by the version of the UPDRS-ME developed by the Movement Disorder Society (MDS-UPDRS-ME^13^,^15^), of which the Dutch translation is validated in our centre. The MDS-UPDRS-ME hand items used in this study include finger tapping (thumb on index finger), rapid hand opening/closing movements and pro/supination of the hand for assessment of bradykinesia.^13^,^15^ The scale ranges from 0 to 4, with 0 being normal and a higher score indicating more pronounced symptoms. Tremor was assessed during rest with and without a mental task, during a posturing task (holding hands outstretched below chin) and during an active kinetic task (moving index finger between patient’s nose and the finger of the examiner). The wrist rigidity test consisted of passive wrist flexion/extension (performed by the examiner) with and without contralateral activation by making a fist.

### PowerGlove Procedure

Besides the clinical assessment, all patients were evaluated with the PowerGlove system. The order of the clinical assessments by nurse and the PowerGlove assessment was randomized.

At the start of PowerGlove measurements, an anatomical calibration procedure was performed on every patient to determine the sensor-to-segment coordinate systems. The calibration procedure included several steps: (i) hand placed on a flat surface with wrist in 0° flexion, (ii) thumb placed on a flat surface, (iii) thumb flexed 3 times in the interphalangeal (IP) joint, (iv) fingers flexed 3 times in the metacarphophalangeal (MCP) joints (fingers stretched, hand still), and (v) hands placed together while performing an eight-shaped movement. From these, 3D segment coordinate systems could be determined which describe the segment orientation of the finger segments and the hand.^21^,^37^ Furthermore, to enable accurate measurements of fingertip positions the lengths of the hand and finger segments were measured using a ruler, which were used for scaling in the applied biomechanical hand model.^21^,^37^ Besides the anatomical calibration, a magnetic field mapping was performed to account for any disturbances caused by e.g. ferromagnetic materials in the direct environment.

To measure the moment applied on the wrist during the rigidity tasks, a force/moment sensor (ATI mini45, ATI Industrial Automation USA) was used. During this measurement, the lower arm was resting on the arm support of the chair. The hand was passively moved by the examiner, while stabilizing the lower arm. The force/moment sensor was attached to the hand of the examiner using a strap. Moment arm of the sensor to the patient’s wrist was measured using a ruler.

Examples of all the PowerGlove assessments are shown in Fig. 1.

### Data Acquisition

Sensor data of the PowerGlove were captured with a sample frequency of 100 Hz using custom-made, Matlab-based software that computed the anatomical segment calibration, and collected information from gyroscopes, accelerometers and magnetometers, and applied this in an extended Kalman filter algorithm that merged all sensory inputs into a biomechanical hand model.^21^ Force data was captured with a sample frequency of 512 Hz *via* a Porti system with PolyBench software (Twente Medical Systems International B.V. Oldenzaal, NL).* Post-hoc*, the force data were synchronized with the PowerGlove data based on an external synchronization signal that was visible in the magnetometer data of the PowerGlove and in a separate channel captured *via* the same Porti system as the force data. For this, the data from the force sensor and the synchronization signal were down-sampled to 100 Hz.

### Data Analysis and Parameters

**Figure 2:**
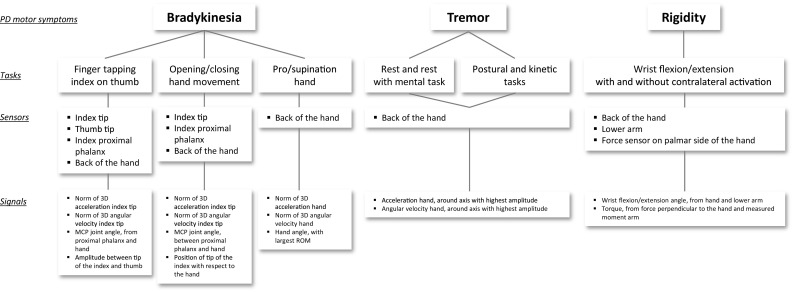
Sensors and signals used to determine hand motor symptoms in Parkinson’s disease with the PowerGlove system.

The signals measured and computed by the PowerGlove system could be used for quantification of each of the PD hand motor symptoms. These signals include angular velocity and acceleration of the hand and finger segments, positions of segments (e.g. fingertip), joint angles (e.g. MCP joint) and applied force (for the rigidity test only) (Fig. 2). From these signals, many outcome parameters can be extracted to quantify PD motor symptoms (Table 2). The selection of these parameters is based on the parameters previously used in the literature (Table 1) and on characteristics of the assessed phenomena, such as movement information for bradykinesia and tremor (e.g. amplitude, velocity and frequency characteristics) and movement and force information for rigidity (e.g. torque–angle relations) (Fig. 2; Table 2). To demonstrate the applicability of the PowerGlove system for the quantification of PD hand motor symptoms, differences in the outcome parameters listed in Table 2 were explored in off- vs. on-medication condition.

**Table 2:** Investigated parameters for quantification of bradykinesia, rigidity and tremor obtained by signals measured with the PowerGlove system.

Task	Parameters
Bradykinesia	
Finger tapping index on thumb Hand opening/closing	RMS of the acceleration of the index fingertip, over all cycles (norm of 3D) RMS of the angular velocity of the index fingertip, over all cycles (norm of 3D) Time of movement intervals (i.e. cycle time) using MCP angle, average and standard deviation of all cycles Minimum, maximum and ROM of the MCP angle of the index finger, average and standard deviation of all cycles (flexion angle) Minimum, maximum and ROM of the distance (i.e. amplitude) between index and thumb fingertips, average and standard deviation of all cycles (finger tapping) Minimum, maximum and ROM of the distance (i.e. amplitude) between index fingertip and hand, average and standard deviation of all cycles (hand open/close)
Pro/supination hand	RMS of acceleration of the hand, over all cycles (norm of 3D) RMS of angular velocity of the hand, over all cycles (norm of 3D) Time of movement intervals (i.e. cycle time) using the hand angle, average and standard deviation of all cycles Minimum, maximum and ROM of the hand angle, average and standard deviation of all cycles (angle with largest ROM)
Tremor	
During rest During mental task During postural task During kinetic task	RMS of acceleration of the hand (around axis with highest acceleration) RMS of angular velocity of the hand (around axis with highest angular velocity) Peak power in de tremor band (4–10 Hz), using the acceleration of the hand Total power in the tremor bands (4–10 Hz), using the acceleration of the hand
Rigidity	
Wrist flexion/extension (passive)	Maximal ROM of wrist angle Torque at 20° and 50° wrist extension at wrist joint Maximal torque Stiffness of wrist joint (10–90% range of motion window) Impulse of wrist joint (10–90% time window) Work of wrist joint (10–90% range of motion window)

*RMS* root means square, *ROM* range of motion, *MCP* metacarphophalangeal joint

#### Bradykinesia

Bradykinesia during finger tapping was quantified using data of four sensors of the PowerGlove: the sensors on the tip of the index finger, tip of the thumb, proximal phalanx of the index finger, and the back of the hand (Fig. 2). Since movement could occur around all three axes of a sensor, the norm of the accelerations in three directions (3D: x,y,z) and the norm of the 3D angular velocities were calculated. MCP joint angles (flexion/extension) and 3D index and thumb fingertip positions were obtained from the PowerGlove software based on the anatomical calibration, forward kinematics and biomechanical hand model.^21^ The distance between the tips of the index finger and thumb was calculated by the norm of the difference between the two position vectors in space. The time of each movement interval was determined using the MCP angle (cycle time, average and standard deviation over cycles). For acceleration and angular velocity, the root mean square (RMS) over all cycles was calculated. Furthermore, for the MCP angle and thumb/index amplitude, the minimum, maximum and range of motion (ROM) per cycle was determined, and consequently the average and standard deviation over cycles were calculated as outcome parameters (Table 2).

For analysis of hand opening/closing movements, data of three sensors of the PowerGlove were used: the sensors on the tip of the index finger, proximal phalanx of the index finger, and hand (Fig. 2). The same procedure was followed as for the finger tapping task, including similar parameters (Table 2). Instead of index/thumb amplitude, the trajectory of the index fingertip position with respect to the hand was calculated using the norm of the vector.

For pro/supination, data of the hand sensor were used (Fig. 2). In line with the procedure as described above, the norm of the hand accelerations in three directions (3D: x,y,z) and the norm of the 3D hand angular velocities were calculated. For the other outcome parameters based on the hand angle (Fig. 2; Table 2), first the 3D hand angles in the global coordinate system were determined in which the angles were calculated with respect to the anatomical reference position (hand and lower arm placed on a flat surface). Subsequently, from the 3D hand angles, the hand angle with the greatest ROM was selected as representing the pro/supination angle.

#### Tremor

Tremor was quantified using data of the hand sensor (Fig. 2). Data of accelerometers and gyroscopes were bi-directional second-order high-pass filtered at 1.0 Hz (Butterworth)^1^,^11^,^16^,^18^ to remove slow voluntary movements and dyskinesia. For the kinetic task, the acceleration and angular velocity data were bi-directional second-order high-pass filtered at 4.0 Hz (Butterworth) to also remove the faster voluntary movement.

Spectral analysis (discrete Fourier transform) was performed on the 3D acceleration and 3D angular velocity signals to analyse the data in the frequency domain. Peak power and total power around the axis with the highest amplitude was evaluated in the 4–10 Hz frequency band, i.e. the band in which PD tremor can be expected for both rest and kinetic tasks, therewith excluding voluntary movement (<4 Hz) and physiological tremor (10–12 Hz).^1^,^2^,^6^,^11^,^16^,^20^,^32^ The total power of the tremor band was calculated by numerical integration of the power in the frequency bands. Also, RMS values of acceleration and angular velocity around the axis with the highest amplitude were calculated (Table 2).

#### Rigidity

Wrist rigidity was quantified using the inertial sensors on the lower arm and hand, and the force sensor that was placed on the palmar side of the patient’s hand by the examiner during the wrist extension/flexion movement (Fig. 2). The wrist flexion/extension angle was calculated from the angle between lower arm and hand, anatomically calibrated using a posture with hand and lower arm on a flat surface in 0° flexion/extension. The torque (i.e. wrist joint moment) was calculated by multiplying the measured moment arm with the force measured perpendicular to the hand. For analysis, only the wrist extension movements were selected. Selected wrist movements were averaged for further calculation of parameters. For maximal ROM, the maximal extension angle was determined. Torque was defined at 20 and 50° wrist extension and at maximal wrist extension. Stiffness was defined as the slope of the torque–angle curve of the wrist, over the 10–90% range of motion window (in degrees). Impulse was calculated by the integration of torque over a 10–90% time window (in seconds) and work was determined by integration of torque over angle using the 10–90% range of motion window (in degrees) (Table 2).

## Results

**Figure 3:**
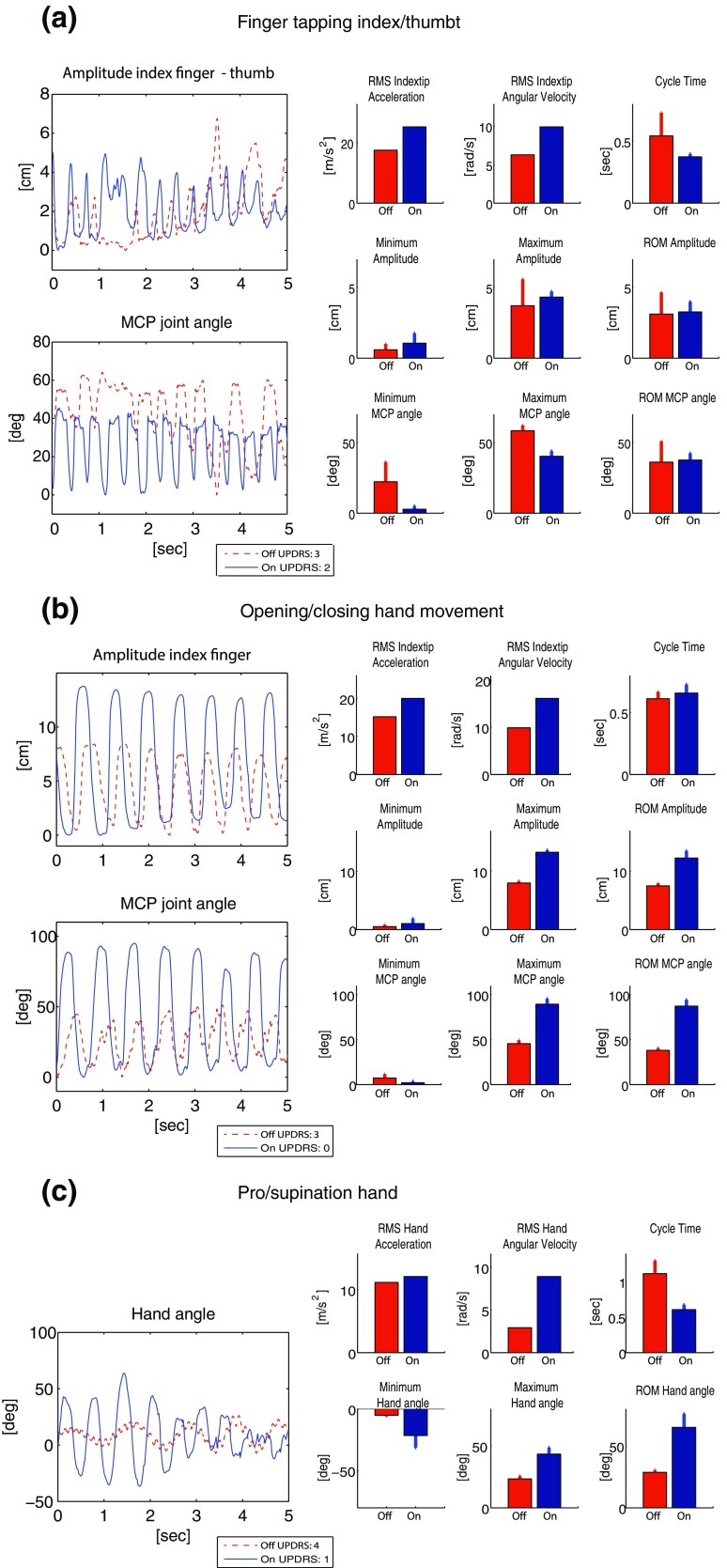
Bradykinesia of the hand in Parkinson’s disease patients. Outcome parameters measured with the PowerGlove in off- and on-medication condition: (a) finger tapping (patient 1); (b) opening/closing hand movements (patient 2); (c**)** pro/supination of the hand (patient 2). For all parameters, the average per cycle (barplot) and the standard deviations over cycles are shown. For RMS, the value of the whole time window is shown. MDS-UPDRS-ME scores are shown in the legends.

**Figure 4:**
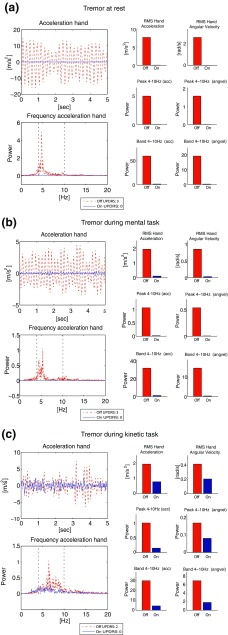
Tremor of the hand in Parkinson’s disease patients. Outcome parameters measured with the PowerGlove in off- and on-medication condition: (a) tremor at rest (patient 3); (b) tremor during mental task (patient 1); (c) Tremor during kinetic task (patient 2). The frequency band (4–10 Hz) is indicated with the black dotted lines. MDS-UPDRS-ME scores are shown in the legends.

**Figure 5:**
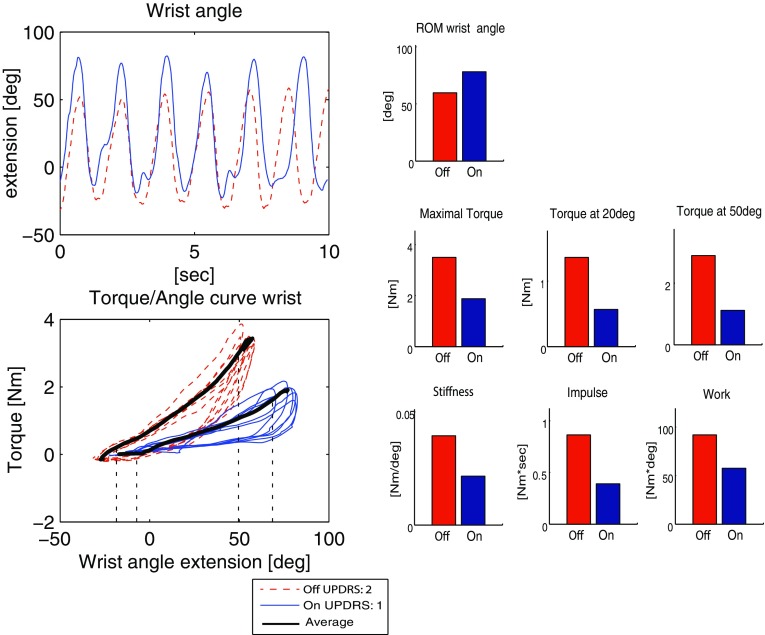
Rigidity of the wrist in a Parkinson’s disease patient. Outcome parameters measured with the PowerGlove in off- and on-medication condition (patient 4). The 10–90% range of motion windows are indicated with the black dotted lines. MDS-UPDRS-ME scores are shown in the legends.

All patients were able to perform the anatomical calibration of the PowerGlove and the tasks related to the hand in the MDS-UPDRS-ME with the PowerGlove attached, in both off- and on-medication condition. The total time to measure all hand motor symptoms varied between 15 and 30 min per condition. Typical examples of data and outcome parameters of the four representative patients measured with the PowerGlove in off- vs. on-medication condition are presented in Figs. 3, 4, and 5 for bradykinesia, tremor and rigidity respectively, showing a difference between conditions in most outcome parameters. MDS-UPDRS-ME scores of these patients are shown in the legends.

### Bradykinesia

Finger tapping (Fig. 3a; UPDRS off: 3, on: 2) shows higher movement speed in on- vs. off-medication condition. This is most expressed in the increased RMS acceleration and RMS angular velocity and in the decreased cycle time. The minimal MCP joint angle was lower in the on- vs. off-medication condition, which means that the index finger was in general more extended during movement in the on-condition. Full MCP joint extension (0°) was not always reached in off-medication condition. Most pronounced was the movement irregularity in off-medication condition, which is expressed in high standard deviations of cycle time, amplitude (maximum and ROM) and MCP angle (minimum and ROM). The average ROM values did not show clear differences.

Opening/closing hand movement (Fig. 3b; UPDRS off: 3, on: 0) shows clear improvement in all parameters in the on-medication condition (i.e. increased velocity, acceleration, amplitude and range of motion), except for the cycle time. The minimum MCP angle shows good extension in both conditions. This example does not show any hesitation in the presented time-frame in either off- or on-medication condition; the cycle time standard deviations are small in both conditions.

The pro/supination parameters (Fig. 3c; UPDRS off: 4, on 1) show improvement in all parameters in on- vs. off-medication condition, i.e. a higher velocity, shorter cycle time and larger ROM. In the hand angle it can be observed that pro/supination movement in off-medication condition could barely be performed, as seen from the small ROM. Furthermore, over the slow pro/supination movements in off-medication condition, a tremor movement (of about 7 Hz) can be observed.

### Tremor

Parameters for tremor at rest (UPDRS off 3, on 0) and tremor during a mental task (UPDRS off 3, on 0) and a kinetic task (UPDRS off 2, on o) show a difference in all parameters in on- vs. off-medication condition (Fig. 4). No tremor is present in on-medication condition during rest and during the mental task, whereas in off-medication condition tremor is clearly present as can be seen by the large power in the 4–10 Hz frequency band, where tremor is expected for PD patients. Furthermore, a high RMS is seen in off-medication condition (related to high amplitude, present most of the time).

### Rigidity

Finally, the quantification of parameters of the wrist rigidity task (Fig. 5, without contralateral activation; UPDRS off 2, on 1) shows that due to the medication intake, the wrist ROM is increased, whereas torque, stiffness, impulse and work are all decreased in comparison to the off-medication condition.

## Discussion

### Applicability of the PowerGlove and Proposed Outcome Parameters

In the past, several measurement systems have been proposed to quantify the hand motor symptoms in PD. However, only a few studies have attempted to measure at least two of the phenomena of bradykinesia, tremor and rigidity with a single system or together in one study.^8^,^31^,^32^,^35^ In our study, the PowerGlove enabled us to measure all hand motor symptoms in PD patients using a combination of inertial sensors and a force sensor, where the force sensor was only applied for the assessment of wrist rigidity. We selected a variety of outcome parameters to show the applicability of the PowerGlove (Table 2), to measure differences in off- vs. on-medication condition and to explore optimal outcome parameters.

Previously, it has been shown that for assessment of bradykinesia, increased angular velocity (maximum, RMS) is related to improvement after DBS^8^,^20^ and can be used to discriminate between UPDRS-ME scores.^18^,^23^,^35^ Furthermore, amplitude (distance between the tips of the index finger and thumb) has been shown to be sensitive in measuring differences in bradykinesia, such as after DBS.^8^

In the finger tapping task, a large and fast index finger movement is important, and full extension of the MCP joint needs attention. Not all parameters showed improvement in on-medication condition. In line with literature, RMS of angular velocity and acceleration as well as maximum amplitude did improve in the given example (i.e. increased in on-medication condition). Furthermore, average cycle time and standard deviations of cycle time and ROM did decrease. However, average ROM of amplitude and MCP angle did not clearly discriminate on- and off-medication condition. That is likely caused by irregularity of the movement in off-medication condition, where fast and small movements are followed by several slow and large movements (in accordance to a MDS-UPDRS-ME score of 3^15^), leading to a mean ROM that is comparable to the on-medication state. Because a patient’s movement in the off-medication state can be affected in different ways (see e.g. the patterns described in Ref. 3), it remains important to always combine mean values with standard deviations when interpreting ROM values.

In the pro/supination task, improvement was observed in all measured parameters of the presented patients. Also for hand opening/closing movement, all parameters improved, except cycle time.

The different results of the three bradykinesia tasks illustrate that bradykinesia can improve in different ways for different tasks and/or different patients, either as an improvement in amplitude, speed or both. For example, in the opening/closing movement, an increase in amplitude can cloud an increase in speed, when only measuring cycle times. An objective and quantitative measurement system like the PowerGlove enables us to measure different aspects of hand motor control that might be clinically relevant for PD patients. In the clinical exam, this might be difficult to observe and will surely be difficult to separately quantify using only the 0–4 scoring system. Quantifying only one parameter might be too restricted to describe the complex phenomenon of bradykinesia, since this is related to several aspects of the movement like amplitude, ROM, velocity, occurrence of hesitation and mean and SD of the time of each cycle. Yet, the PowerGlove system enables us to combine several quantitative parameters.

For tremor, literature indicates that peak power^16^ and RMS and amplitude of acceleration and angular velocity^1^,^24^,^32^ are correlated to clinical scores or discriminative in off- vs. on-medication. We did not include amplitude for tremor, but RMS was calculated for acceleration and angular velocity around the axis with highest amplitude and showed differences between off- and on-medication in the presented example. The same was true for all power parameters. It is likely that tremor can be quantified fairly easily by many systems, therefore, the added value of the PowerGlove mainly lies in the ability to combine tremor measurement with bradykinesia and rigidity measurements.

Finally, for the assessment of rigidity, in literature torque,^7^,^8^ impulse^10^,^22^ and the viscous damping constant^22^,^26^,^34^ were shown to correlate with the UPDRS-ME scores, improve after DBS or differentiate between patients and controls, whereas work was shown not to be a valid measure.^10^,^26^ We measured increased ROM and decreased torque, stiffness, impulse and work, reflecting a reduction in rigidity in the PD patient. Viscous damping constant has not been calculated. This parameter has to be determined using a fitting spring-damper model and reflects velocity-dependent behavior.^22^,^26^ Such an approach is more complex and, since all chosen parameters improved after medication intake, it is questionable whether including this parameter would be necessary to quantify a difference in on- and off-medication state.

Since in the rigidity test the ROM in on-medication condition is increased, this might have influenced the calculated work, and possibly also the impulse calculations. Using a similar window in the on- and off medication condition, e.g. the ROM of the off-medication condition, would further reduce the calculated work in on-medication condition. The use of the PowerGlove might enable standardization of either the measured or analyzed ROM between on- and off-medication measurement, which might increase the reliability of rigidity measurements. However, the maximal ROM itself is also an outcome that could be clinically relevant for PD patients.

### Future Directions

This proof-of-principle study is the first step in a larger study, in which the aim is to test the validity and reliability of the proposed outcome parameters in a larger cohort of PD patients. For validity, the correlation of the outcome parameters with the MDS-UPDRS-ME scores could be further studied. However, not all proposed parameters might be suitable to discriminate between small deviations in a patient’s clinical condition, or could also be redundant with respect to each other. Therefore, per clinical phenomenon an optimal set of parameters should be defined describing the different aspects of that particular motor symptom (as discussed above), which could guide clinical evaluation and decision-making. Additionally, evolution of parameters over the course of one measurement can be of importance. An optimal set of parameters to describe different aspects of a motor symptom could be found by using methods such as machine learning. This may lead to the development of a prediction tool with classification algorithms based on measurement sets to e.g. predict MDS-UPDRS-ME scores, or to discriminate small changes in the outcome parameters that cannot be observed with the clinical score.

The influence of reattachment of sensors and a second anatomical calibration in between conditions on the outcome parameters needs to be further investigated in an intra- and inter-rater reliability study. This calibration is important for the determination of the MCP joint angle, hand angle and fingertip positions. Results of a previous study on a comparison between the PowerGlove and an opto-electronic system (a standard in most movement analysis laboratories) already give some insights in the effect of calibration.^37^ It shows that the effect of another anatomical calibration (in that case due to using a different measurement system) is limited to a difference in finger joint angles between 3° and 8° and a difference in thumb/index amplitude of less than 16 mm. The on- vs. off-differences presented in the PD patients (Figs. 3, 4, 5) are larger than these differences.

For determination of the outcome parameters, not all data of all sensors have been used (Table 2). Therefore, for future application of the PowerGlove in a clinical setting, a reduced set of sensors might be sufficient to obtain clinically relevant outcome parameters. In a recent study, only sensors on the tip of the index finger and thumb are proposed to measure angular changes during finger tapping.^3^ However, to estimate distance between the tip of the index finger and of the thumb in bradykinesia tasks, a reduced set of sensors is only applicable with the prerequisite that position of the tip of the index finger and thumb can be modeled without any information about the orientation of the middle phalanx of the index and the metacarpal and proximal phalanges of the thumb (such as in Nataraj *et al.* 25). Data of the middle finger is not used at all, and these sensors could therefore easily be omitted.

For easy attachment to the fingers, the miniature inertial sensors should be embedded in either a glove or easy to use straps connected to the sensors. With the current straps, the sensors were firmly attached, but a quicker way of attachment is desirable. Currently, the system is not wireless. However, the wires are lightweight and patients mentioned no limitations in their movement because of the wires. Still, for future application, a wireless system would be desirable. Moreover, the sensors/glove should be easy to clean, especially when future applications during DBS surgery are envisioned, in which quantitative assessment of symptom severity could guide DBS implantation or optimization of stimulation settings. If a next version of the system will be developed for future DBS applications, using for example a glove approach, new studies on reliability and validity are needed, in which current knowledge on the clinical applicability and outcome parameters, such as presented in this paper, will be helpful. Until now, only a few studies attempted to quantify motor symptoms during DBS surgery^17^,^20^ or for automatic optimization of DBS settings.^31^ Optimization of DBS settings can be a challenge due to the number of variables that must be considered, including presence of multiple motor signs, side effects and battery life.^31^ A simple and objective way to quantify the motor symptoms in the hand can definitively assist in this challenging process.

### Limitations of the Study

Outcome parameters are calculated over a time-window of 5 s. Although this time frame is sufficient to illustrate the applicability, longer time-windows may be necessary to assess certain aspects of PD hand motor symptoms, such as hesitation in movement or decrease in amplitude over time for bradykinesia tasks. Also, when a patient shows large fluctuations in tremor symptoms, longer registration and the detection of tremor and non-tremor windows might be beneficial.

For determination of outcome parameters in the rigidity task, only the force perpendicular to the palmar side of the hand has been used. In this way, the calculation of the torque has been simplified. Contribution of forces and moments in other directions, as well as the contribution of mass and inertia of the hand and the force sensor itself, have been neglected. It was assumed that the force applied to the hand was largest in the direction of the movement. For mass parameters (mass, inertia and center of gravity), an anthropometric model is required of the hand. Since the hand is a small structure, contribution of these parameters were assumed to be negligible.

Finally, since this is a proof-of-principle study, the number of subject included and the number of examples shown is limited. Therefore, no group results and statistics could be presented. In a future study, a larger cohort of patients will be included and analyzed to assess the clinimetric properties of the PowerGlove and to derive to an optimal set of parameters per symptom.

## Conclusion

The methodology described and the results presented in the current paper show the applicability of the PowerGlove to objectively quantify bradykinesia, tremor and rigidity in PD patients in a clinical research setting. The presented examples showed a difference in off- vs. on-medication condition in all tasks for most outcome parameters. Further study into validity and reliability of the proposed outcome parameters is required in a larger group of patients, to arrive at an optimal set of parameters that will guide clinical evaluation and decision making.
